# Comparing the Assembly and Handedness Dynamics of (H3.3-H4)_2_ Tetrasomes to Canonical Tetrasomes

**DOI:** 10.1371/journal.pone.0141267

**Published:** 2015-10-27

**Authors:** Rifka Vlijm, Mina Lee, Orkide Ordu, Anastasiya Boltengagen, Alexandra Lusser, Nynke H. Dekker, Cees Dekker

**Affiliations:** 1 Department of Bionanoscience, Kavli Institute of Nanoscience, Delft University of Technology, Delft, The Netherlands; 2 Division of Molecular Biology, Biocenter, Innsbruck Medical University, Innsbruck, Austria; National Cancer Institute, UNITED STATES

## Abstract

Eukaryotic nucleosomes consists of an (H3-H4)_2_ tetramer and two H2A-H2B dimers, around which 147 bp of DNA are wrapped in 1.7 left-handed helical turns. During chromatin assembly, the (H3-H4)_**2**_ tetramer binds first, forming a tetrasome that likely constitutes an important intermediate during ongoing transcription. We recently showed that (H3-H4)_2_ tetrasomes spontaneously switch between a left- and right-handed wrapped state of the DNA, a phenomenon that may serve to buffer changes in DNA torque induced by RNA polymerase in transcription. Within nucleosomes of actively transcribed genes, however, canonical H3 is progressively replaced by its variant H3.3. Consequently, one may ask if and how the DNA chirality dynamics of tetrasomes is altered by H3.3. Recent findings that H3.3-containing nucleosomes result in less stable and less condensed chromatin further underline the need to study the microscopic underpinnings of H3.3-containing tetrasomes and nucleosomes. Here we report real-time single-molecule studies of (H3.3-H4)_2_ tetrasome dynamics using Freely Orbiting Magnetic Tweezers and Electromagnetic Torque Tweezers. We find that the assembly of H3.3-containing tetrasomes and nucleosomes by the histone chaperone Nucleosome Assembly Protein 1 (NAP1) occurs in an identical manner to that of H3-containing tetrasomes and nucleosomes. Likewise, the flipping behavior of DNA handedness in tetrasomes is not impacted by the presence of H3.3. We also examine the effect of free NAP1, H3.3, and H4 in solution on flipping behavior and conclude that the probability for a tetrasome to occupy the left-handed state is only slightly enhanced by the presence of free protein. These data demonstrate that the incorporation of H3.3 does not alter the structural dynamics of tetrasomes, and hence that the preferred incorporation of this histone variant in transcriptionally active regions does not result from its enhanced ability to accommodate torsional stress, but rather may be linked to specific chaperone or remodeler requirements or communication with the nuclear environment.

## Introduction

The DNA in eukaryotic nuclei is substantially compacted by histone-induced packaging. The basic unit of compaction is the nucleosome: 147 base pairs (bp) of DNA that are wrapped 1.7 times around a histone octamer [1, 2]. The octamer is built up of two copies each of the core histones H2A, H2B, H3 and H4. All four families of core histones are highly positively charged with a conserved C-terminal histone fold domain and unique N-terminal tails [3]. The histone fold domains interact strongly with the other core histones within the nucleosome, and with the nucleosomal DNA. The tails do not display significant intra-nucleosomal contacts, but instead interact with neighboring nucleosomes and other proteins (e.g. remodelers). Nucleosomes are stabilized by the opposite charges of the histones and DNA backbone, but do not spontaneously assemble at physiological salt conditions [4]. Binding of histones to DNA occurs in a prescribed order, with each step being facilitated by a chaperone loading protein: first the (H3-H4)_2_ tetramer binds to the DNA to form a tetrasome, and then two H2A-H2B dimers are added to form the complete nucleosome. The (H3-H4)_2_ tetramer, also relevant in the biological context e.g. as a result of H2A-H2B dimer loss during transcription [5–9], has a horseshoe-shaped structure that includes an H3-H3 interface at its center [1, 10].

Packaging of DNA into nucleosomes affects the accessibility of DNA in important processes like transcription and replication. As such, the structure, number, position, and stability of nucleosomes impact multiple nuclear processes. To regulate DNA accessibility, chromatin remodeling proteins can assemble and evict nucleosomes, alter nucleosome position, or induce structural changes to histones or histone replacement [11]. Additionally, there exist many histone variants, which, play specific roles either through their unique positioning on the genome [3, 12–14] or by acting during specific phases of the cell cycle, serve as a ‘toolbox for genome regulation’.

**Fig 1:**
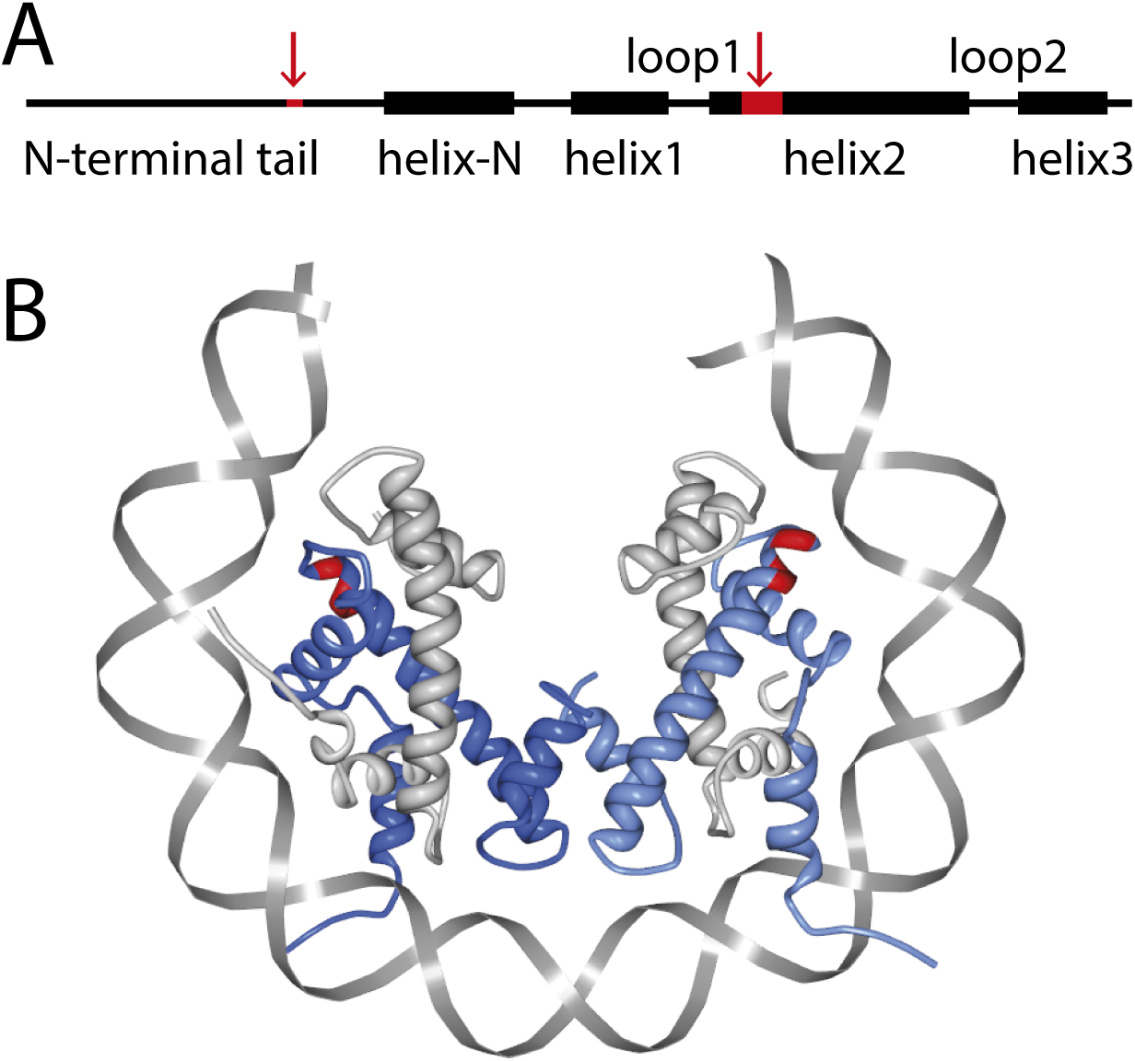
Domains and illustration of the (H3.3-H4)_2_ tetrasome. (A) The Drosophila H3 and H3.3 vary by only four amino acids that are marked in red. One is located in the N-terminal tail domain and three are located in the α-helix 2 domain, at locations 87, 89 and 90 (after [3]). (B) The crystal structures for H3-H4 and H3.3-H4 tetrasomes are not known, but to illustrate the most likely configuration, we here show a visual rendering of the structure of the human nucleosome containing the histone variant H3.3 (3AV2 from PDB). Only histones H3.3 (blue) and H4 (grey) are shown, as well as part of the nucleosomal DNA (grey). The amino acid locations 87, 89 and 90 are marked in red to indicate the region that deviates from the canonical histone H3 in the histone fold domain. The amino acid variant in the N-terminal tail is not marked, since it is outside of this region. This image was created using the software described in Ref. [43].

The *Drosophila melanogaster* genome encodes three H3 histone variants. These are the canonical H3 (H3.2 [15]) histone, which is only expressed during S phase when the DNA is replicated; the main replacement histone H3.3 [16, 17], which differs from H3 by only four amino acids; and the CENP-A (CID) variant, which is only located in centromeric regions and structurally deviates more from the other H3 histone variants [18]. Focusing specifically on the differences between H3.3 and canonical H3, three of the differing amino acids lie in α-helix 2 of the histone fold domain, while the fourth lies in the N-terminal region (**Fig 1**; [3]). Unlike H3, H3.3 is not restricted to S phase but is instead expressed and loaded onto chromatin throughout the entire cell cycle, predominantly at transcriptionally active regions [19]. Additionally, H3.3 has recently been located at silent chromatin loci such as telomeres and centromeres [20], and it appears to be required for male fertility [21]. It has been shown that replacement of any one of the three different amino acids in the histone-fold domain of H3 by the corresponding H3.3 counterpart results in replication-independent deposition of the histone [19]. Since the three histone-fold domain amino acids are located at the surface of the α-helix 2 domain that is responsible for the formation of H3-H4 dimers and accessible in prenucleosomal complexes [22, 23], it has been suggested that the specificity associated with these amino acid positions derives from interactions with different assembly or post-translational-modification machineries. From a structural perspective, however, nucleosomes that contain canonical H3 or H3.3, appear to be very similar [23]. These findings highlight the importance of subtle differences between H3 and H3.3 and call for studies of potential underlying mechanistic differences.

We recently demonstrated that canonical (H3-H4)2 tetrasomes are highly dynamic [7], finding that they exhibit spontaneous flipping between a preferentially occupied left-handed DNA wrapping and a less favored right-handed wrapping. Only upon addition of H2A/H2B is the left-handed state locked in and are full nucleosomes formed [7]. The handedness-flipping mechanism was proposed to involve a rotation of the two H3/H4 dimers with respect to each other at the H3-H3 interface, thus making H3 one of the key players in this process [10]. The remarkable torsional flexibility of tetrasomes led us to propose that tetrasomes might function as a twist reservoir under conditions of torsional stress, such as during transcription and replication [9].

During transcription, histone H3.3 (as opposed to H3) is incorporated into nucleosomes. To support transcription, chromatin fibers containing H3.3 nucleosomes tend to be less condensed [19, 24–26]. We aim to investigate whether tetrasomes and nucleosomes containing H3.3 have a different structure and structural stability compared to canonical tetrasomes/nucleosomes. Possibly, H3.3 tetrasomes/nucleosomes are less compacted, and any significant energy barrier between the left- and right-handed tetrasome states or an increased overall preference for a right-handed state could hinder the formation of left-handed nucleosomes. Using Freely Orbiting Magnetic Tweezers (FOMT; [27]) we directly monitored both the real-time NAP1-mediated assembly of individual (H3.3-H4)_2_ tetrasomes onto bare DNA and the subsequent dynamics. For further characterization, we also investigated the torsional response of tetrasomes using Electromagnetic Torque Tweezers (eMTT; [28]). We find that the changes in DNA compaction and chirality upon assembly of H3.3-H4 containing tetrasomes and nucleosomes by the histone chaperone Nucleosome Assembly Protein 1 (NAP1) occur in a manner identical to that of H3-containing tetrasomes and nucleosomes. Likewise, the flipping behavior of H3.3-containing nucleosomes is similar to that of canonical tetrasomes. Flushing out free NAP1 and histones in solution only slightly enhanced the positively wrapped state of the tetrasome.

## Materials and Methods

### Single-molecule Instrumentation

The traces monitoring NAP1-assisted nucleosome and tetrasome assembly in real time via changes in extension and linking number, as well as any subsequent dynamics in linking number, were measured using the FOMT [27]. The torque measurements were carried out using the eMTT [28]. All measurements were performed at 21°C and acquired at an acquisition frequency of 100 Hz.

### Protein expression and purification

Recombinant *Drosophila* core histones were expressed in *E*. *coli* BL21(DE3) Rosetta (Novagen) and purified as described in [29], with the distinction that the purification procedure for the H3.3-H4 tetramers was identical to that of the H2A/H2B dimers. Expression plasmids were a kind gift of J. Kadonaga. Concentrations of core histones were determined by SDS PAGE and Coomassie staining as well as calculated from A_280_ measurements and the H3-H4 extinction coefficient (**S1 Fig**). Recombinant *Drosophila* NAP1 was purified according to [30].

### Flow cell passivation and buffer conditions

In all experiments, we employed a buffer consisting of 50 mM KCl, 25 mM Hepes-KOH pH 7.6, 0.1 mM Ethylenediaminetetraacetic acid (EDTA), 0.025% Polyethylene Glycol (PEG) and 0.025% Polyvinyl Alcohol (PVOH) as crowding agents, and 0.1 mg/ml bovine serum albumin (BSA) both as crowding agent and for the prevention of nonspecific binding of histones to the surface. For the tetrasome assembly we used the histone chaperone NAP1. Although *in vivo* NAP1 is known as a histone chaperone for H2A and H2B, *in vitro* it has been shown that NAP1 assembles complete nucleosomes [7, 31–35]. The used protein concentrations were: 200 nM NAP1, 67 nM H3.3 and 67 nM H4 were preincubated for 30 min on ice. The pre-incubation buffer contained 50 mM KCl, 25 mM Hepes pH 7.6, 0.1 mM EDTA, 0.25% PEG, 0.25% PVOH and 1 mg/ml BSA. Just prior to flushing in, the protein concentration was reduced ~4000-fold. To achieve nucleosome assembly following tetrasome formation, 270 nM NAP1, 268 nM H2A, 268 nM H2B were preincubated for 30 min on ice. Just before flushing in, the protein concentration was reduced ~300-fold.

### DNA constructs

We used 1.9 kilo-base-pair (kbp) double-stranded DNA (dsDNA) molecules in the FOMT experiments and 3.4 kbp DNA molecules in the eMTT experiments, both without positioning sequences (sequence available in the S1 File). To attach the DNA molecules to the glass surface and the bead, their extremities contained multiple digoxigenin molecules at one end and multiple biotin molecules at the other end. The DNA molecules did not contain any nucleosome-positioning sequences. In the FOMT experiments, we used 0.5 μm diameter beads (Ademtech) and in the eMTT experiments we used 0.7 μm diameter beads (MagSense).

## Results and Discussion

### NAP1-assisted assembly of (H3.3-H4)_2_ tetrasomes

**Fig 2:**
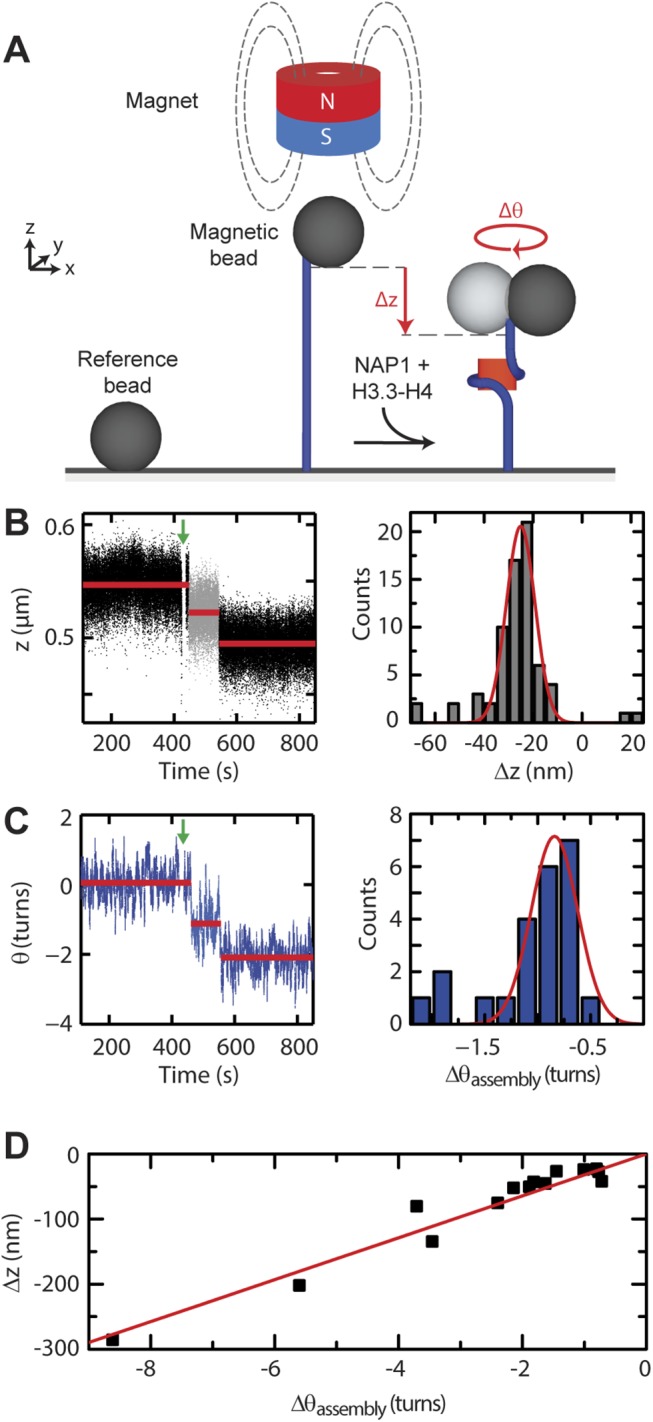
NAP1-assisted (H3.3-H4)_2_ tetrasome assembly. (A) Schematic of the *in vitro* assay showing a single DNA molecule (blue) tethered between a glass surface and a paramagnetic bead. The circular magnet above the bead applies a stretching force to the bead (and hence to the DNA), but leaves it free to rotate about the DNA-tether axis. A nonmagnetic reference bead is fixed to the surface to allow for drift correction. After flushing in NAP1 preincubated with histones H3.3-H4, tetrasomes are loaded onto the DNA. (B) Time-dependence of the end-to-end length *z* (μm) (left) of a single DNA tether during the assembly of two (H3.3-H4)_**2**_ tetrasomes. The step sizes are -25 and -27 ± 5 nm. The green arrow at *t* = 420 s indicates the flushing in of the proteins. Data was acquired at 100 Hz, and red lines indicate the mean values of each assembly step. The histogram on the right derives from 19 independent assembly experiments (69 steps). A Gaussian fit shows that the average step in *z* during tetramer assembly is -25 ± 6 nm. (C) Time-dependence of bead rotations *θ* (turns) (left) of the same DNA tether as in B). Compaction of the DNA (shown in B)) occurs concurrently with a change in linking number (changes in *θ*). The step sizes in *θ* are -1.17 ± 0.24 and -0.97 ± 0.24 turns. The green arrow at *t* = 420 s indicates the flushing in of the proteins. Data was acquired at 100 Hz, and red lines indicate the mean values of each assembly step. The histogram on the right derives from 15 independent assembly experiments (23 steps). It can be fitted to Gaussian peaks. The most likely step in *θ* during tetramer assembly is -0.8 ± 0.1 turns. A small number of steps appears to result from the simultaneous assembly of two tetramers, with a mean step size in *θ* of -1.9 ± 0.1 turns. (D) The total degree of compaction (*Δz*) plotted versus the total change in linking number (*Δθ*_assembly_) on 25 individual DNA molecules following the assembly of tetrasomes (black squares). Fits to a linear relationship yield *Δz/Δθ*_assembly_ = 32 ± 2 nm (solid red line).

We directly monitored tetrasome formation upon flushing in H3.3 and H4 pre-incubated with the histone chaperone NAP1 into the flow cell using FOMT, a technique that allows one to simultaneously measure dynamical changes in the end-to-end length and linking number of single tethered DNA molecules. In this approach, a vertically oriented magnetic field is used to apply a stretching force, without constraining the free rotation of the DNA molecule (**Fig 2A**). The DNA molecules employed (1.9 kbp in length) did not contain specific nucleosome-positioning sequences. We limited the applied stretching force to 0.8 pN, well below the 3 pN above which DNA begins to peel off from the nucleosome [36]. Upon flushing in NAP1/histone complexes, this experimental configuration allowed us to observe a distinct, stepwise decrease in the end-to-end length *z* of the DNA, indicating compaction, accompanied by a clockwise rotation *θ* of the bead, reflecting a decrease in the linking number of the DNA tether (left panels in **Fig 2B and 2C**). From several independent (H3.3-H4)_2_ assembly experiments, we obtained an average extension change <Δ*z*> = -25 ± 6 nm (**Fig 2B**, right) and linking number change <*Δθ*_assembly_> = -0.8 ± 0.2 turns (**Fig 2C**, right). By changing the histone concentration, we assembled varying numbers of tetrasomes per DNA molecule. The total degree of compaction Δ*z* and the overall change in linking number Δ*θ*_*assembly*_ following assembly were found to be linearly correlated with a slope Δ*z*/Δ*θ*_*assembly*_ of 32 ± 2 nm/turn (**Fig 2D**).

These single-molecule assembly experiments revealed that NAP1 is capable of assembling (H3.3-H4)_2_ tetrasomes. The obtained average extension change <Δ*z*> = -25 ± 6 nm for assembly of (H3.3-H4)_2_ tetrasomes agrees well with previous results on canonical tetrasomes and nucleosomes [7, 35, 36], indicating that the alterations in the histone fold domain of H3.3 do not affect the overall tetrasome structure formed. The linear correlation between the total degree of compaction Δ*z* and the overall change in linking number Δ*θ*_*assembly*_ following assembly indicated that the conformation of the tetrasomes on the DNA is independent of the number of protein complexes assembled, excluding large effects of inter-tetrasomal interactions.

### (H3.3-H4)_2_ tetrasomes exhibit spontaneous dynamic changes in linking number

**Fig 3:**
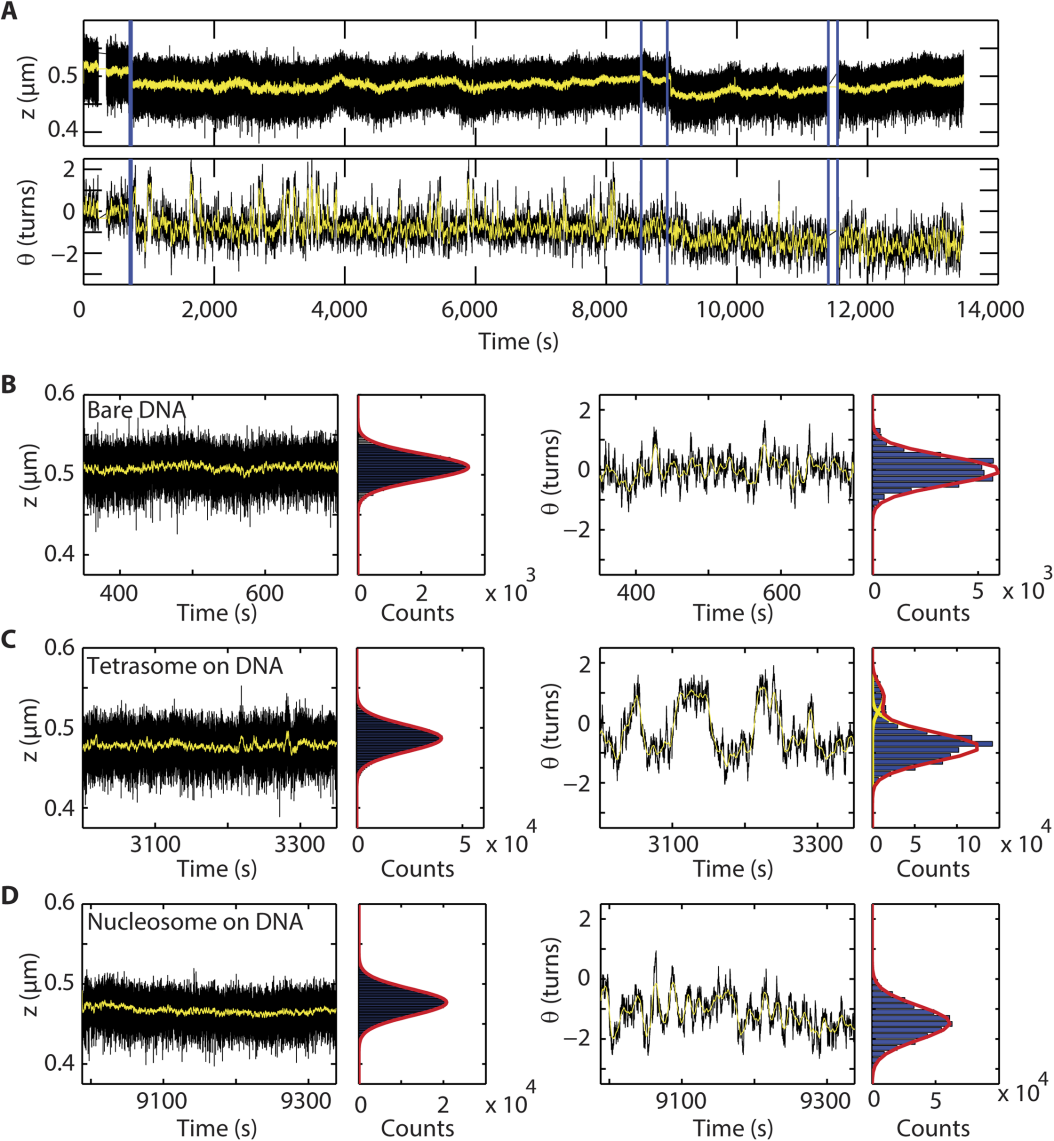
(H3.3-H4)_2_ tetrasomes undergo dynamic changes in linking number and form a viable intermediate for nucleosomes. (A) Assembly of a single complete nucleosome from a single assembled tetrasome. By flushing in H3.3-H4 preincubated with NAP1 at *t* = 708 s, we assembled one tetrasome (*Δz* = 23 ± 5 nm, *Δθ*_*assembly*_ = -0.81 ± 0.25 turns). Dynamic changes in the linking number were observed immediately following assembly and continued for ~8000 s. When we then flushed in histones H2A and H2B preincubated with NAP1 at 8536 and 8935 s, we observed an additional assembly step (*Δz* = 31 ± 5 and *Δθ* = -0.55 ± 0.25 turns). Subsequently, the linking number remained stable (i.e. the flipping behavior of the handedness ceased). Blue lines mark flushing in of NAP and core histones H3.3-H4 at *t* = 708 s and of H2A-H2B at *t* = 8536 s and *t* = 8935 s. All proteins are flushed out at *t* = 11400 s. Parts of the data shown in A) are highlighted in panels B)—D). The left panels show a typical segment (350 s) of the end-to-end length *z* (left) and the angular coordinate *θ* (right). Side panels show histograms with fits to Gaussian functions (red lines) that are derived from the full portion of the trace acquired under the indicated conditions. (B) Bare DNA, before the proteins are flushed in. (C) DNA loaded with a single tetrasome. The centers of the Gaussian fits are at -0.80 and 0.86 turns. (D) DNA loaded with a single nucleosome. In B-D), the mean extension, *z*, remains constant in time, with fluctuations merely arising from Brownian motion (standard deviations of σ_bare DNA_, σ_tetrasome_, and σ_nucleosome_ are 23 nm). Both bare DNA and DNA loaded with nucleosomes exhibit a fixed mean linking number in time, with comparable fluctuations about the mean (σ = 0.66 and 0.77 turns, respectively). However, tetrasomes exhibit clear fluctuations in the linking number over time.

We also carried out a separate set of experiments to determine whether there were further dynamics to be observed on the single-molecule tethers following assembly. For reference, bare DNA has a constant length *z* and linking number *θ*, apart from Brownian fluctuations (**Fig 3A and 3B**). Following the assembly of a single (H3.3-H4)_2_ tetrasome (which compacted the DNA by 22 ± 1 nm), the resulting reduced DNA length *z* stayed constant (**Fig 3A and 3C** left panels). Concomitant with the step in *z*, a step in the linking number of -0.81 ± 0.25 turns occurred (**Fig 3A and 3C** right panels). However, subsequently the linking number did not stay constant but was rather observed to change between -0.80 ± 0.05 and +0.86 ± 0.39 turns (**Fig 3A and 3C** right panels). From this, we concluded that (H3.3-H4)_2_ tetrasomes exhibited spontaneous flipping between a preferentially occupied left-handed state and a right-handed state in a manner that left the DNA extension unchanged. On average, the (H3.3-H4)_2_ tetrasome showed spontaneous fluctuations in the linking number with a typical *ΔL*_*k*_ = 1.68 ± 0.14 turns, a mean change in linking number very similar to that observed for the canonical tetrasome [7].

To determine whether this flipping tetrasome could accommodate the assembly of a complete nucleosome, we subsequently added histones H2A-H2B (**Methods**). This led to a decrease in the mean linking number by 0.55 ± 0.25 turns in a single step, together with an arrest of the flipping behavior (**Fig 3D**, right panel). The total amount of compaction due to the assembly of the H2A-H2B was 31 nm. Adding histones H2A-H2B thus led to the assembly of a left-handed nucleosome with a total linking number of -1.36 ± 0.2 turns and total compaction of 54 ± 7 nm. We find that the handedness of nucleosomes containing only H3.3 remained stable.

**Fig 4:**
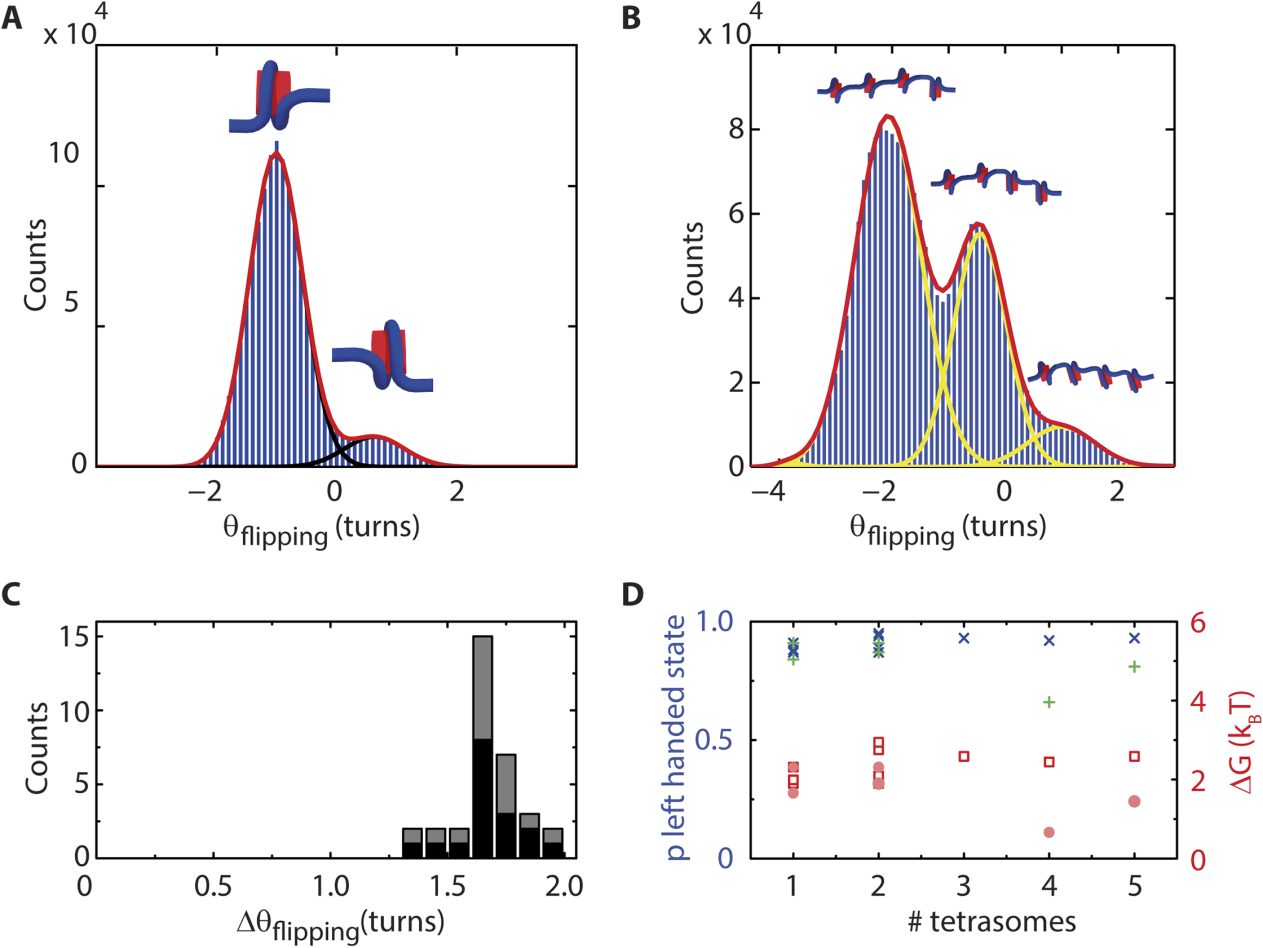
Analysis of *θ*_*flipping*_ of (H3.3-H4)_2_ tetrasomes. (A) A single (H3.3-H4)_**2**_ tetrasome (for a different molecule than that of **Fig 3**, to emphasize repeatability). The histogram of *θ*_flipping_, the difference in angle between the left- and right-handed states from a single (H3.3-H4)_**2**_ tetrasome, shows two peaks. The peak has a maximum at *θ* = -1.011 ± 0.003 turns. The positively wrapped state has a peak at *θ* = 0.63 (± 0.03) turns. (B) Histogram of *θ*_*flipping*_ of a DNA molecule loaded with four tetrasomes. Data are collected after flushing out free proteins. The most pronounced peaks are for 1 (*θ* = -2.1 turns), 2 (*θ* = -0.44 turns) and 3 (*θ* = 1.0 turns) tetrasomes in the right-handed state (values extracted from Gaussian fitting to the histogram). When any one tetrasome flipped to the right-handed state, the linking number increased on average by 1.7 ± 0.2 turns. (C) Histogram of dynamical linking number steps observed following assembly of tetrasomes on distinct DNA molecules (N = 10) before (black) and after (grey) flushing out free proteins (N = 33), which yields a mean value of <Δ*θ*_*flipping*_> = 1.7 ± 0.1 turns both before and after flushing out of free proteins. (D) Determination of the probability p of finding a tetrasome in the left-handed state in the presence (N = 12, dark blue crosses, <*p*> = 0.91 ± 0.03) and absence (N = 7, green plusses, <*p*> = 0.84 ± 0.09) of free proteins. Using the formula ΔG = -k_B_T ln((1/p)-1), the difference in the free energy between the two states can be computed (red datapoints). We deduce ΔG = 2.3 ± 0.4 k_B_T prior to flushing out free proteins (red open squares) and ΔG = 1.6 ± 0.8 k_B_T following the flushing out of free proteins (red filled circles).

We observed flipping signatures in the linking number for every DNA molecule that was loaded with H3.3-containing tetrasomes (but never for bare DNA nor for nucleosome-loaded DNA). A second example of a single tetrasome is shown in **Fig 4A**, whereas the behavior of four assembled tetrasomes, together with cartoons illustrating the number of tetrasomes in the left- and right-handed configuration, is shown in **Fig 4B**. To exclude any potential effect of NAP1 on the dynamics of (H3.3-H4)_2_ tetrasomes, we also performed an experiment in which we removed the free proteins. Under these conditions, DNA molecules with tetrasomes, both for conditions with (black) and without (grey) free proteins in solution, displayed similar-sized angular steps between the discrete levels, <*Δθ*_flipping_ > = 1.6 ± 0.1 turns (**Fig 4C**). These results, taken together with a similar independence of flipping dynamics on NAP1 observed for canonical tetrasomes assembled by salt dialysis [7], leads us to conclude that NAP1 does not induce the change in handedness of the (H3.3-H4)_2_. Collectively, these experiments show that the inherent flipping behavior of the handedness of tetrasomes is not limited to H3-containing tetrasomes, but also applies to tetrasomes that contain H3.3. Moreover, they demonstrate that the dynamically flipping (H3.3-H4)_2_ tetrasome is a viable intermediate in the assembly of stable, left-handed, nucleosomes.

The flipping of the canonical tetrasomes loaded onto DNA by NAP1 can be analyzed in the framework of a binomial model in which a single tetrasome occupies either the left- or right-handed state with probabilities *p* and (1-*p*), respectively [7]. A value of *p* close to 1 indicates that tetrasomes are much more likely to occupy the left-handed state over the right-handed state, whereas a shift towards lower values of *p* indicates a more positively wrapped tetrasome. For each experiment, we determined the relative occupancies of each state from the ratios of the respective peak areas in the linking number histograms. We fitted *p* for distinct DNA molecules loaded with different numbers of tetrasomes resulted in a <*p*> = 0.91 ± 0.03 (*N* = 12) in the presence of free proteins (**Fig 4D,** dark blue crosses). Using *ΔG = -k*_*B*_ *T ln (1/p– 1)* to compute the free energy difference between the left- and right-handed states, we deduced a free energy difference between the left- and right-handed states of 2.3 ± 0.4 *k*_B_ *T* (D, dark red squares), similar to the 2.3 *k*_B_ *T* value found for canonical tetrasomes and the 2.5 *k*_B_ *T* value determined via electrophoretic mobility analysis of nucleosome populations [5, 7]. We note that that we measured a slightly reduced probability for the occupancy of the left-handed state <*p*> = 0.84 ± 0.09 (*N* = 7) after flushing out free proteins (**Fig 4D,** green plus signs), corresponding to a decreased free energy difference between the states of 1.6 ± 0.8 *k*_B_ *T* (**Fig 4D**, filled pink circles). Flushing out of the proteins thus mildly increases the probability to occupy the right-handed state of the (H3.3-H4)_2_ tetrasome. This finding, together with the observation that *Δθ*_*flipping*_ is unaffected by the removal of free proteins, suggests that NAP1 may stimulate the left-handed wrapping slightly while leaving the linking number of the left- and right-handed states unchanged.

### Minute torques can drive structural transitions within (H3.3-H4)_2_ tetrasomes

**Fig 5:**
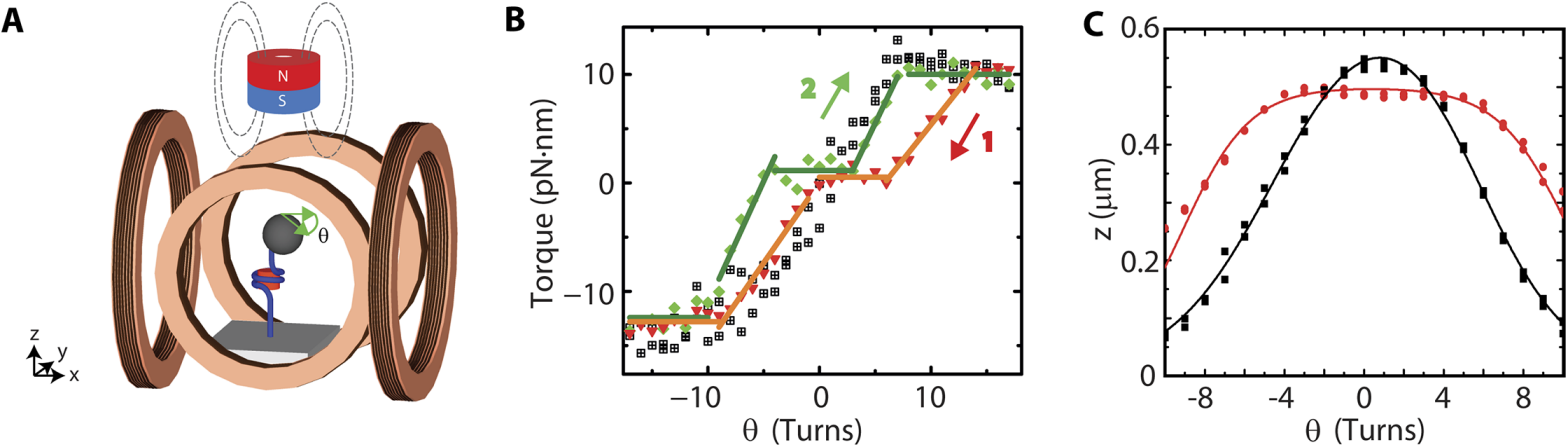
Torque response of DNA loaded with (H3.3-H4)_2_ tetrasomes. (A) Diagram of the eMTT configuration used in these experiments. The eMTT resembles the FOMT configuration, but additionally has two pairs of Helmholtz coils placed around the flow cell to permit the application of torque in the horizontal plane. (B) The torque stored in DNA loaded with 5 tetrasomes plotted as a function of the number of applied rotations, *θ*. The black squares represent the data for a bare DNA molecule, prior to assembly. Following assembly, the torque response of DNA loaded with tetrasomes is measured by decreasing the number of applied turns from +17 to -17 (red triangles, labeled by ‘1’). Consecutively, the torque response of DNA loaded with tetrasomes is measured in the opposite direction by increasing the number of applied turns -17 to +17 (green diamonds, labeled by ‘2’). The solid lines are segmented fits to the plateau regions (with slope 0) and to the sloped regions in which torque is built up. The widths of the plateaus for positive (red) and negative (green) rotation directions are 7.5 ± 1 and 5.9 ± 1 turns, respectively, as determined from the intersections between the segmented fits. The applied force is ~0.8 pN. (C) The DNA end-to-end length plotted as a function of the number of rotations, *θ*. The applied stretching force is 0.3 pN. The black squares show the data for a bare DNA molecule, prior to any tetrasome assembly. Following tetrasome assembly, a broad plateau (of ~8 ± 1 turns) is observed surrounding 0 turns (red circles). The solid lines are linear fits to the data (5 per trace).

We next studied the response of (H3.3-H4)_2_ tetrasomes to physiologically relevant applied torques [37] at an applied stretching force of 0.8 pN by using eMTT [28] (**Fig 5A**). For these experiments, we utilized 3.4 kbp DNA (again without specific nucleosome-positioning sequences) loaded with tetrasomes by NAP1. Reference measurements on bare DNA showed that the application of turns to torsionally relaxed bare DNA initially left the DNA extension unchanged as the DNA twist increased, resulting in a linear build-up of torque (black squares, **Fig 5B**). At a critical buckling torque, a decrease in the DNA extension *z* was observed as DNA buckled to form plectonemic supercoils, and beyond this, no further torque build-up occurred (plateau in black squares for > 6 turns and < -6 turns in **Fig 5B**). The torque response following NAP1-mediated assembly of ~5 (H3.3-H4)_2_ tetrasomes (deduced from the total length decrease of 135 nm given the average length decrease of 25 nm per tetrasome, **Fig 2B**) is shown in **Fig 5B**. Starting at positively induced supercoiling, the torque response was first measured from +17 turns to -17 turns (red triangles). Consecutively, the measurement direction was reversed (green diamonds). At the center of both torque response curves, a plateau at nearly zero torque was clearly visible. In this region, the induced turns did not lead to build up of twist (and hence torque) in the tethered molecule; instead, changes in the tetrasome conformation likely occurred that prevented such build-up. Alternately stated, a negligibly low torque could be used to drive a tetrasome into a left-handed configuration (when negative turns were imposed) or into a right-handed configuration (when positive turns were imposed). The widths of the near-zero torque plateaus for the negative (red) and positive (green) rotation directions were 7.5 ± 1.0 and 5.9 ± 1.0 turns, respectively (**Fig 5B**). Therefore the ∆L_k_/tetrasome in the plateaus is 1.5 and 1.2 for the negative and positive rotation direction respectively. Once a sufficient number of turns was applied to force all tetrasomes to occupy either left- or right-handed states, torque build-up ensued as in the case of bare DNA. Finally, saturation of torque build-up occurred beyond a torque of +10 (-12) pN nm (accompanied by a concomitant decrease in extension, consistent with plectoneme formation), also similar to the case of bare DNA.

We briefly examine the regions in which torque is built up for DNA loaded with tetrasomes (**Fig 5B**). In all cases, these slopes are shallower than those measured for bare DNA; this could reflect gradual changes in the conformation of the loaded tetrasomes. An example of this would be a change in the angle of the tetrasome’s entry and exit DNA. Additionally, the torque response curves display hysteresis: neither the slopes of the torque response nor the widths of the plateaus around zero rotations are identical upon reversal of the direction of rotation. From the constant length of the molecule (data not shown), we can conclude that this hysteresis is not induced by tetrasome dissociation/rebinding events. Instead, it appears that the induced conformational changes from right- to left-handed tetrasomes is more gradual than vice versa. For example, for the molecule shown in **Fig 5**we expect that ~5 tetrasomes have been assembled as deduced from the length decrease upon assembly. We therefore expect the width of the zero-torque plateau to comprise 5 x (*θ*_*flipping*_ =) 1.7 = 8.5 turns **Fig 4C**). Given the measured plateau widths of 7.5 turns (from negative to positive rotations) and 5.9 turns (from positive to negative rotations), it appears that 0.2–0.5 turns per tetrasome are absorbed by more gradual conformational changes that occur as the magnitude of the torque in the tetrasome-loaded DNA is decreased. Summing up, these experiments directly demonstrate that the application of only very weak positive torques can drive tetrasomes from left- into right-handed states. Furthermore, the torque response displays hysteresis as a function of the direction of rotation, indicating that the sudden conformational changes as shown by handedness flipping at low torques (e.g., conformational change at the H3.3-H3.3 interface as suggested in (18)) are accompanied by more gradual conformational changes (e.g., due to slight changes in direction of the entry and exit DNA) under the influence of torque) at increased levels of torque.

## Discussion and Conclusions

The influence of DNA topology, specifically transcription-induced supercoiling, on gene regulation is an emerging topic of interest. It has been suggested that nucleosome assembly and disassembly processes, through their modification of the local degree of supercoiling, can play important roles in gene regulation on distances exceeding several kb [38–42]. In this research, we have found that canonical (H3.1-H4)_2_ and variant (H3.3-H4)_2_ tetrasomes exhibit similar behavior in both their assembly and subsequent dynamical changes in linking number. A comparison of all measured parameters for these two types of tetrasomes is shown in Table 1. Furthermore, both types of tetrasomes are viable intermediates for nucleosomes. We find that the overall number of assembled (H3.3-H4)_2_ tetrasomes does not affect the change in linking number per tetrasome under our experimental conditions, from which we conclude that the assembly is not affected by inter-nucleosomal interactions. Once assembled, (H3.3-H4)_2_ tetrasomes exhibit flipping in their chirality under the influence of thermal fluctuations that is similarly independent of the number of assembled tetrasomes and comparable to the case of canonical tetrasomes. The ease of flipping tetrasome handedness is also displayed by the appearance of a near-zero torque plateau in the torque response of DNA assembled with tetrasomes, similar to the previously studied (H3-H4)_2_ tetrasomes. The additional hysteresis in the torque-turns curve indicates that mild gradual changes to the tetrasome structure also occur. The collective similarity with the results obtained for canonical (H3-H4)_2_ tetrasomes demonstrates that the incorporation of H3.3 does not change the biophysical properties of tetrasomes. Therefore, the presence of H3.3 in transcriptionally active regions does not signal an enhanced ability to accommodate torsional stress, but may rather be linked to specific chaperone or remodeler requirements or communication with the nuclear environment.

**Table 1:** Comparison of the key physical properties measured for tetrasomes composed of (H3-H4)_2_ (left; Ref. [7]) versus (H3.3-H4)_2_ (right; this work).

	(H3-H4)_2_	(H3.3-H4)_2_
**Δ*z***	-24 ± 3 nm	-25 ± 7 nm
**Δ*θ***_***assembly***_	-0.73 ± 0.05 turns	-0.8 ± 0.1 turns
**Δ*z* (total) / Δ*θ***_***assembly***_ **(total)**	34 ± 1 nm/turn	32 ± 2 nm/turn
**Δ*θ***_***flipping***_	1.7 ± 0.1 turns	1.7 ± 0.1 turns
**P before flush out**	0.9 ± 0.08	0.91 ± 0.03
**P after flush out**	-	0.84 ± 0.09
**Binomial distribution**	Yes	Yes
**Viable nucleosome intermediate**	Yes	Yes
**Minute torques can drive structural transitions**	Yes	Yes

## Supporting Information

S1 Fig Protein gel of the used H3.3 and H4 histones. The gel indicates 140 μg/ml of each histone.
(TIF)

S1 File DNA sequences of the used fragments. (PDF)
