# Supplementary figures and images for: Comparing the Assembly and Handedness Dynamics of (H3.3-H4)2 Tetrasomes to Canonical Tetrasomes
Source: PLoS One. 2015 Oct 27;10(10):e0141267. doi: 10.1371/journal.pone.0141267 (PMC4623960; doi:10.1371/journal.pone.0141267)

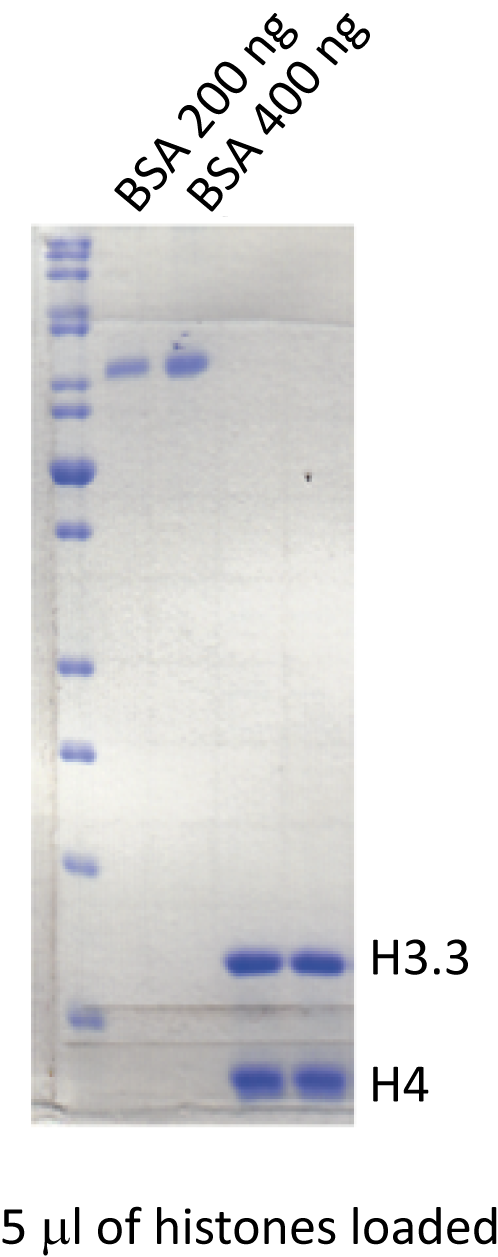

Supplement: S1 Fig — The gel indicates 140 μg/ml of each histone. (TIF) [file pone.0141267.s001.tif]
