# Supplementary material for: Comparing the Assembly and Handedness Dynamics of (H3.3-H4)2 Tetrasomes to Canonical Tetrasomes
Source: PLoS One. 2015 Oct 27;10(10):e0141267. doi: 10.1371/journal.pone.0141267 (PMC4623960; doi:10.1371/journal.pone.0141267)
Supplement: S1 File — (PDF) [file pone.0141267.s002.pdf]

DNA sequence 1.9 kb fragment:

TGCCGTTGTAACCGGTCATCCCCGAGTACGGCTGCAGCGCCCGCGTCCGGCTGACCAGCGTGCCGGA
CACCGGCAGCACGGCGATGCCGTTCATGACCTGATAACTGCGGGCCTGTCGTGGTCCGTCATCATCA
CCGGATAATGCCAGCGTCGCGAGTGCCTCCTGGGCAGTCAGGCTGTCGCCGGACACCGCATCCGTCA
GGCTGCTGATCCCAAGCTGGCCTGCAAGCGCACAAAAGAAAACCCGCGCATAGGCGGGTTCAAGCA
TCAGCGGCTCATTAAAGGCCATGCTGGCAATATGCGGGAGATTACGCAGCTCTGCTGTCACTCTTCTC
CTCCTCTGTTGATTGTCGCAGCCCGGATTCAAATGCTGCAGCCGCCCAGGCGGGCGGTTTAAGACCG
GCTGCACGGCGCTCCATCGTTTCACGGACCTGCTGGGCAAAAATTTCCTGATAGTCGTCACCGCGTTT
TGCGCACTCTTTCTCGTAGGTACTCAGTCCGGCTTCTATCAGCATCACCGCTTCCTGAACTTCTTTCAG
ACCATCGATGGCCATACGACCGGAGCCTATCCAGTCGCAGTTCCCCCAGGCACTGCGGGCTTCCTGA
AAACTGAAGCGCGCTTTTGAAGGTAACGTCACCACGCGGCGAACGATGGCCTCTTCCAGCCAGCACA
GAAACATCTGGCTCGCCTGACGGGATGCGACGAATTTTCGCCGCCCCATAAAGTACGCCCACGACTC
GTTCGCACTGGCCCGTGCCGTGGAGTAGCTCATCTGGGCGTAATTCCGGGAAAGCTGCTCATACGAG
ACACCCAGCCCGGCAGCGATATACCGCAGCAGTGACTGCTCAAACACGGAGTAGCCGTTATCCGTAT
CCTGAGCCGTCTGCAGGTTCAGTGAGTCACCCGGCATCAGGTGCGGTACTTTTGCGCCTCCCAGCCG
GACCGGCGCTGCGGCGTAATACGCGGCAATTTCACCAATCCAGCCGGTCAGCCTTTCCCGCTGCTCCT
GACTGTTCGCGCCCAGAATAAAATCCATCGCTGACTGCGTATCCAGCTCACTCTCAATGGTGGCGGC
ATACATCGCCTTCACAATGGCGCTCTGCAGCTGCGTGTTCTGCAGCGTGTCGAGCATCTTCATCTGCT
CCATCACGCTGTAAAACACATTTGCACCGCGAGTCTGCCCGTCCTCCACGGGTTCAAAAACGTGAATG
AACGAGGCGCGCCCGCCGGGTAACTCACGGGGTATCCATGTCCATTTCTGCGGCATCCAGCCAGGAT
ACCCGTCCTCGCTGACGTAATATCCCAGCGCCGCACCGCTGTCATTAATCTGCACACCGGCACGGCA
GTTCCGGCTGTCGCCGGTATTGTTCGGGTTGCTGATGCGCTTCGGGCTGACCATCCGGAACTGTGTC
CGGAAAAGCCGCGACGAACTGGTATCCCAGGTGGCCTGAACGAACAGTTCACCGTTAAAGGCGTGC
ATGGCCACACCTTCCCGAATCATCATGGTAAACGTGCGTTTTCGCTCAACGTCAATGCAGCAGCAGTC
ATCCTCGGCAAACTCTTTCCATGCCGCTTCAACCTCGCGGGAAAAGGCACGGGCTTCTTCCTCCCCGA
TGCCCAGATAGCGCCAGCTTGGGCGATGACTGAGCCGGAAAAAAGACCCGACGATATGATCCTGAT
GCAGCTGGATGGCGTTGGCGGCATAGCCGTTATTGCGTACCAGATCGTCTGCGCGGGCATTGCCAC
GGGTAAAGTTGGGCAACAGGGCTGCATCCACACTTTCACTCGGTGGGTTCCACGACCGCAACTGCCC
TCCAAATCCGCTGCCACCGCCGTGATAACCGGCATATTCGCGCAGCGATGTCATGCCGTCCGGCCCC
AGAAGGGTGGGAATGGTGGGCGTTTTCATACATAAAATCCTGCAGGTCCCCTGCGTCGCTGAGG

DNA sequence 3.4 kb fragment:

CAGGTGGCACTTTTCGGGGAAATGTGCGCGGAACCCCTATTTGTTTATTTTTCTAAATACATTCAAAT
ATGTATCCGCTCATGAGACAATAACCCTGATAAATGCTTCAATAATATTGAAAAAGGAAGAGTATGA
GTATTCAACATTTCCGTGTCGCCCTTATTCCCTTTTTTGCGGCATTTTGCCTTCCTGTTTTTGCTCACCCA
GAAACGCTGGTGAAAGTAAAAGATGCTGAAGATCAGTTGGGTGCACGAGTGGGTTACATCGAACTG
GATCTCAACAGCGGTAAGATCCTTGAGAGTTTTCGCCCCGAAGAACGTTTTCCAATGATGAGCACTTT
TAAAGTTCTGCTATGTGGCGCGGTATTATCCCGTATTGACGCCGGGCAAGAGCAACTCGGTCGCCGC
ATACACTATTCTCAGAATGACTTGGTTGAGTACTCACCAGTCACAGAAAAGCATCTTACGGATGGCAT
GACAGTAAGAGAATTATGCAGTGCTGCCATAACCATGAGTGATAACACTGCGGCCAACTTACTTCTG
ACAACGATCGGAGGACCGAAGGAGCTAACCGCTTTTTTGCACAACATGGGGGATCATGTAACTCGCC
TTGATCGTTGGGAACCGGAGCTGAATGAAGCCATACCAAACGACGAGCGTGACACCACGATGCCTG
TAGCAATGGCAACAACGTTGCGCAAACTATTAACTGGCGAACTACTTACTCTAGCTTCCCGGCAACAA
TTAATAGACTGGATGGAGGCGGATAAAGTTGCAGGACCACTTCTGCGCTCGGCCCTTCCGGCTGGCT

GGTTTATTGCTGATAAATCTGGAGCCGGTGAGCGTGGGTCTCGCGGTATCATTGCAGCACTGGGGCC
AGATGGTAAGCCCTCCCGTATCGTAGTTATCTACACGACGGGGAGTCAGGCAACTATGGATGAACG
AAATAGACAGATCGCTGAGATAGGTGCCTCACTGATTAAGCATTGGTAACTGTCAGACCAAGTTTAC
TCATATATACTTTAGATTGATTTAAAACTTCATTTTTAATTTAAAAGGATCTAGGTGAAGATCCTTTTT
GATAATCTCATGACCAAAATCCCTTAACGTGAGTTTTCGTTCCACTGAGCGTCAGACCCCGTAGAAAA
GATCAAAGGATCTTCTTGAGATCCTTTTTTTCTGCGCGTAATCTGCTGCTTGCAAACAAAAAAACCAC
CGCTACCAGCGGTGGTTTGTTTGCCGGATCAAGAGCTACCAACTCTTTTTCCGAAGGTAACTGGCTTC
AGCAGAGCGCAGATACCAAATACTGTTCTTCTAGTGTAGCCGTAGTTAGGCCACCACTTCAAGAACTC
TGTAGCACCGCCTACATACCTCGCTCTGCTAATCCTGTTACCAGTGGCTGCTGCCAGTGGCGATAAGT
CGTGTCTTACCGGGTTGGACTCAAGACGATAGTTACCGGATAAGGCGCAGCGGTCGGGCTGAACGG
GGGGTTCGTGCACACAGCCCAGCTTGGAGCGAACGACCTACACCGAACTGAGATACCTACAGCGTG
AGCTATGAGAAAGCGCCACGCTTCCCGAAGGGAGAAAGGCGGACAGGTATCCGGTAAGCGGCAGG
GTCGGAACAGGAGAGCGCACGAGGGAGCTTCCAGGGGGAAACGCCTGGTATCTTTATAGTCCTGTC
GGGTTTCGCCACCTCTGACTTGAGCGTCGATTTTTGTGATGCTCGTCAGGGGGGCGGAGCCTATGGA
AAAACGCCAGCAACGCGGCCTTTTTACGGTTCCTGGCCTTTTGCTGGCCTTTTGCTCACATGGCTCGA
CAGATCTGCGCAGCACCATGGCCTGAAATAACCTCTGAAAGAGGAACTTGGTTAGGTACCTTCTGAG
GCGGAAAGAACCAGCTGTGGAATGTGTGTCAGTTAGGGTGTGGAAAGTCCCCAGGCTCCCCAGCAG
GCAGAAGTATGCAAAGCATGCATCTCAATTAGTCAGCAACCAGGTGTGGAAAGTCCCCAGGCTCCCC
AGCAGGCAGAAGTATGCAAAGCATGCATCTCAATTAGTCAGCAACCATAGTCCCGCCCCTAACTCCG
CCCATCCCGCCCCTAACTCCGCCCAGTTCCGCCCATTCTCCGCCCCATGGCTGACTAATTTTTTTTATTT
ATGCAGAGGCCGAGGCCGCCTCGGCCTCTGAGCTATTCCAGAAGTAGTGAGGAGGCTTTTTTGGAG
GCCTAGGCTTTTGCAAAAAGCTTGATTCTTCTGACACAACAGTCTCGAACTTAAGCTGCAGAAGTTGG
TCGTGAGGCACTGGGCAGGTAAGTATCAAGGTTACAAGACAGGTTTAAGGAGACCAATAGAAACTG
GGCTTGTCGAGACAGAGAAGACTCTTGCGTTTCTGATAGGCACCTATTGGTCTTACTGACATCCACTT
TGCCTTTCTCTCCACAGGTGTCCACTCCCAGTTCAATTACAGCTCTTAAGGCTAGAGTACTTAATACGA
CTCACTATAGGCTAGCCACCATGACTTCGAAAGTTTATGATCCAGAACAAAGGAAACGGATGATAAC
TGGTCCGCAGTGGTGGGCCAGATGTAAACAAATGAATGTTCTTGATTCATTTATTAATTATTATGATT
CAGAAAAACATGCAGAAAATGCTGTTATTTTTTTACATGGTAACGCGGCCTCTTCTTATTTATGGCGA
CATGTTGTGCCACATATTGAGCCAGTAGCGCGGTGTATTATACCAGACCTTATTGGTATGGGCAAATC
AGGCAAATCTGGTAATGGTTCTTATAGGTTACTTGATCATTACAAATATCTTACTGCATGGTTTGAACT
TCTTAATTTACCAAAGAAGATCATTTTTGTCGGCCATGATTGGGGTGCTTGTTTGGCATTTCATTATAG
CTATGAGCATCAAGATAAGATCAAAGCAATAGTTCACGCTGAAAGTGTAGTAGATGTGATTGAATCA
TGGGATGAATGGCCTGATATTGAAGAAGATATTGCGTTGATCAAATCTGAAGAAGGAGAAAAAATG
GTTTTGGAGAATAACTTCTTCGTGGAAACCATGTTGCCATCAAAAATCATGAGAAAGTTAGAACCAG
AAGAATTTGCAGCATATCTTGAACCATTCAAAGAGAAAGGTGAAGTTCGTCGTCCAACATTATCATG
GCCTCGTGAAATCCCGTTAGTAAAAGGTGGTAAACCTGACGTTGTACAAATTGTTAGGAATTATAAT
GCTTATCTACGTGCAAGTGATGATTTACCAAAAATGTTTATTGAATCGGACCCAGGATTCTTTTCCAAT
GCTATTGTTGAAGGTGCCAAGAAGTTTCCTAATACTGAATTTGTCAAAGTAAAAGGTCTTCATTTTTC
GCAAGAAGATGCACCTGATGAAATGGGAAAATATATCAAATCGTTCGTTGAGCGAGTTCTCAAAAAT
GAACAATAATTCTAG
